# Comparative assessment of biodegradable-antireflux heparine coated ureteral stent: animal model study

**DOI:** 10.1186/s12894-021-00802-x

**Published:** 2021-02-28

**Authors:** Federico Soria, Julia E. de La Cruz, Juan Pablo Caballero-Romeu, Manuel Pamplona, Daniel Pérez-Fentes, Luis Resel-Folskerma, Francisco M. Sanchez-Margallo

**Affiliations:** 1 Endoscopy-Endourology Department, Jesús Usón Minimally Invasive Surgery Centre Foundation, Carretera N-521, Km. 41.8, C.P.10071 Cáceres, Spain; 2 Endoscopy-Endourology Department, Jesús Usón Minimally Invasive Surgery Centre Foundation, Cáceres, Spain; 3 Urology Department, Alicante University General Hospital, Alicante Institute for Health and Biomedical Research (ISABIAL Foundation), Alicante, Spain; 4 Urology Department, 12 de Octubre University Hospital, Madrid, Spain; 5 University Hospital Santiago de Compostela, Santiago de Compostela, Spain; 6 Urology Department, University Hospital Clínico San Carlos, Madrid, Spain; 7 Jesús Usón Minimally Invasive Surgery Centre Foundation, Cáceres, Spain

**Keywords:** Biodegradable ureteral stent, Heparin coated, Biofilm, Antireflux stents, Morbidity

## Abstract

**Background:**

Double J ureteral stents are widely used on urological patients to provide drainage of the upper urinary tract. Unfourtunately, ureteral stents are not free from complications, as bacterial colonization and require a second procedure for removal. The purpose of the current comparative experimental study is to evaluate a new heparin-coated biodegradable antireflux ureteral stent (BraidStent®-H) to prevent urinary bacterial colonization.

**Methods:**

A total of 24 female pigs were underwent determination of bacteriuria and nephrosonographic, endoscopic and contrast fluoroscopy assessment of the urinary tract. Afterward, were randomly assigned animals to Group-I, in which a 5Fr double-pigtail ureteral stent was placed for 6 weeks, or Group-II, in which a BraidStent®-H was placed. Follow-up assessments were performed at 1, 3, 6, 8, 12 weeks. The final follow-up includes the above methods and an exhaustive pathological study of the urinary tract was accomplished after 20 weeks.

**Results:**

Bacteriuria findings in the first 48 h were significant between groups at 6 h and 12 h. Asymptomatic bacteriuria does not reach 100% of the animals in Group-II until 48 h versus Group-I where it appears at 6 h. The weekly bacteriuria mean rate was 27.7% and 44.4% in Group I and II respectively, without statistical significance. In Group II there were no animals with vesicoureteral reflux, with statistical significance at 3 and 6 weeks with Group-I. The 91.2% of stents in Group-II were degraded between 3 and 6 weeks, without obstructive fragments. Distal ureteral peristalsis was maintained in 66.6–75% in Group-II at 1–6 weeks.

**Conclusions:**

The heparin coating of BraidStent® allows an early decrease of bacterial colonization, but its effectiveness is low at the long term. Heparin coating did not affect scheduled degradation rate or size of stents fragments. BraidStent®-H avoids the side effects associated with current ureteral stents, thus should cause less discomfort to patients.

## Background

Double J ureteral stents are widely used on urological patients to provide drainage of the upper urinary tract [1]. However, these stents are not free from complications and side effects. These mainly include vesicoureteral reflux (VUR), a high rate of bacterial colonisation, incrustation and lower urinary tract symptoms [1, 2]. Furthermore, current ureteral stents require a second procedure for removal. This causes anxiety, an increase in healthcare costs and additional anaesthesia requirements in paediatric patients.

The development of biodegradable ureteral stents (BUS) aims to avoid stent removal and forgotten stent syndrome [3]. In recent years there has been an increase in translational research into biodegradable stents. BUSs are made of several natural and synthetic polymers, with hydrolysis being the main biodegradation process [3–5]. One of the recently described BUS is BraidStent®, which has shown a controlled, predictable biodegradation rate in the porcine model. This stent design is anti-reflux, as it does not interfere with the ureterovesical junction after placement [3, 6]. This represents an important development in the search for the ideal ureteral stent, together with its biodegradable characteristic. However, one of the weaknesses of the BraidStent® was its high asymptomatic bacteriuria rate at 3 weeks of stenting: up to 41%. Heparin coating has shown an ability to reduce bacterial adhesion and incrustation on stent surfaces in previous studies [7, 8], although several authors have disagreed on the ability of the heparin coating to decrease bacterial adhesion [9].

The endpoint of this comparative study was to assess the short- and long-term inhibitory capacity of the novel biodegradable ureteral stent BraidStent®-H in a pig model. This new stent is provided with a heparin coating to avoid the main source of bacterial colonization that occurs at the time of retrograde placement by the introduction of bacteria through the transurethral route in the urinary tract [10] and aims to improve the results achieved with the BraidStent® biodegradable bare stent [3]. Secondary endopoints are related to the evaluation the effect of the heparin coating on the degradation rate, the fragmentation size and excretion of the BraidStent®-H.

## Methods

Twenty-four healthy female pigs were used in this study. The experimental protocol was approved by the Minimally Invasive Surgery Centre´s Ethical Committee for Animal Research (Reference:003/13). This Committee also certifies that the above-mentioned research study was carried out following the guidelines of the of the Animals used for scientific purposes (Directive 2010/63/EU-European Commision). The study was carried out in compliance with the ARRIVE guidelines.

### Phase I: Baseline studies and ureteral stenting

Blood and urine samples were collected to assess blood and biochemical parameters and confirm urine sterility. The urine sample is collected by ultrasound-guided cystocentesis before administration of antibiotic prophylaxis. Next, nephrosonography was performed to assess the degree of upper collecting system dilatation [11] and alterations in the ureteral orifices and bladder trigone [12]. The urothelial alterations at the ureteral orifice and bladder trigone were assessed endoscopically, and categorised according to a validated UOScore (UO_0_: normal ureteral orifice; UO_1_: enlarged ureteral orifice with light surrounding inflammatory reaction; UO_2_: enlarged ureteral orifice with moderate surrounding inflammatory reaction; UO_3_: enlarged ureteral orifice with severe surrounding inflammatory and cystic reaction) [3, 6, 12]. Serum urea and creatinine levels were measured during all study phases.

Simulated voiding cystourethrography (SVCUG) was performed to evaluate VUR at baseline, and at 1, 3, 6, 8 and 12 weeks after ureteral stenting, with a final follow-up at 20 weeks. To perform SVCUG, a Foley catheter was inserted into the urinary bladder and filled. To simulate micturition, the bladder was manually compressed until the pressure reached 50 cm H_2_O for 60 s [3, 6, 12].

Afterwards, excretory urography (EU) was performed to assess upper urinary tract morphology and evaluate ureteral peristalsis under fluoroscopic control (the frequency of ureteral peristalsis from renal pelvis to urinary bladder was evaluated for each animal; waves/min). Finally, the internal lumen of the right ureteropelvic junction (UPJ) was measured by retrograde ureteropyelography to accurately determine the UPJ diameter.

**Fig. 1:**
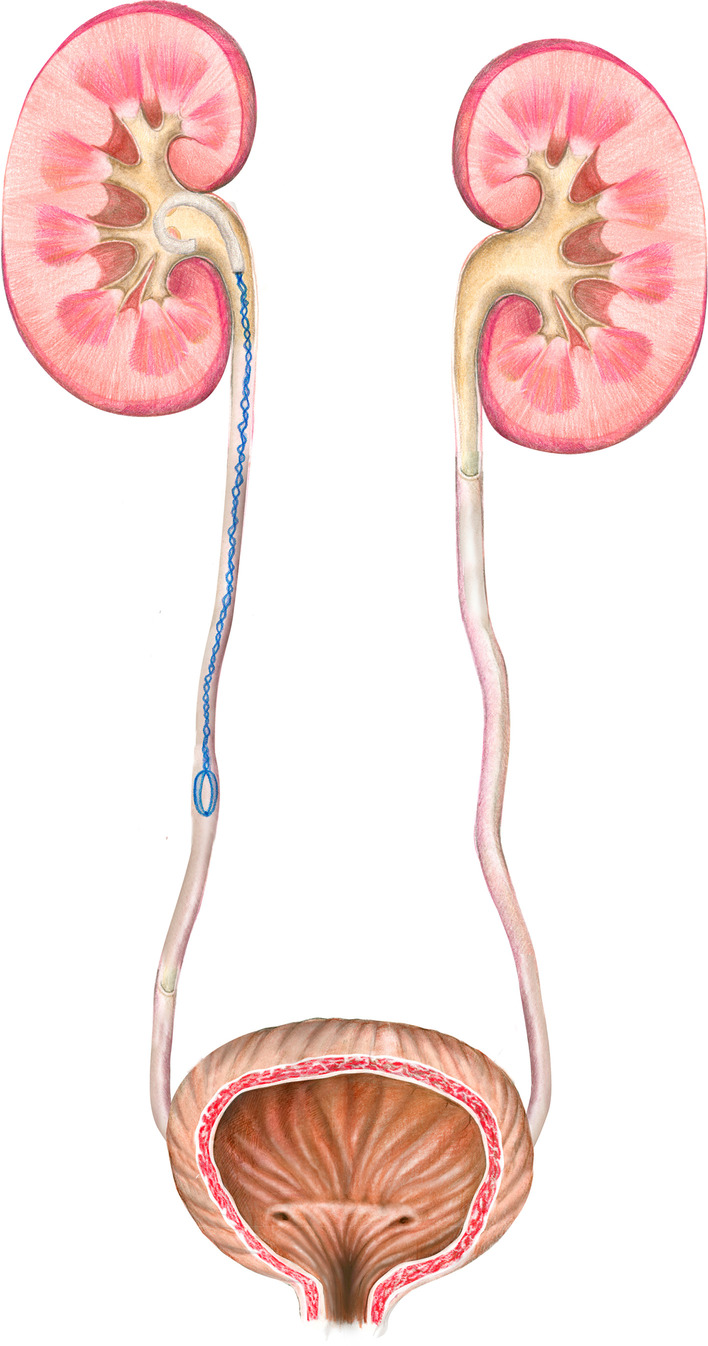
Illustration of the BraidStent®-H in upper urinary tract

Animals were simple randomization distributed into two homogeneous groups:

- Group-I: A 5Fr, 22 cm polymeric ureteral double pigtail stent was placed during a 6-week period (Universa® Soft, 22 cm, Cook® Medical). (Control group).
- Group-II: A new self-retaining, anti-reflux and biodegradable stent, BraidStent-H, was placed using the transurethral approach by sliding over a guide under fluoroscopy control. The placement technique is the same as for conventional stents, with the exception that the pusher is pushed into the ureter*.* BraidStent®-H is an intra-ureteral stent (14 cm length) designed as follows: proximal end with a 3 cm pigtail with an internal channel; a 9 cm-long central section; a four-thread braided section that was 3Fr in diameter; and a distal anchoring system with a rounded edge four-thread basket that measures 2 cm in length and is 36Fr when expanded [3, 6]. Two biodegradable copolymers with different degradation times were used. Polymer-I was Glycomer-631 and polymer-II was pure polyglycolic acid (PGA) [3, 6]. Polymer-II was only used for the central section and was braided with polymer-I. The distal and proximal anchoring systems were manufactured exclusively with polymer-I because it has better biomechanical characteristics and a slower degradation time. This feature allows the stent to remain in place for 3–6 weeks. The BraidStent®-H was designed to avoid passing through the ureterovesical junction (UVJ) to prevent bladder trigone irritation and VUR [3, 6] (Fig. 1).

The Heparin coating of the BraidStent®-H is carried out using the “dip coating” technique. The stents are completely immersed in aluminium containers with sodium heparin with a concentration of 5000 UI/ml (Heparin Hospira 5%, Pfizer Inc, NY, US), in order to be dried afterwards in an oven at 60ºC for 2 h. After this process, each BraidStent®-H has 233 mg of heparin coating. In in vitro studies, we were able to determine the release over time of heparin by the BraidStent-H. The presence and concentration of heparin were analysed by means of an ELISA kit: Human Heparin Sodium ELISA kit MBS3802043MYBIOSOURCE. The heparin coating of the BraidStent®-H starts its release at the beginning of the contact with the urine and is released mainly in the first 72 h.

The onset of asymptomatic bacteriuria was assessed during the first 48 h post-stenting in both study groups in order to evaluate the heparin coating’s ability to inhibit early bacterial colonisation. A urine sample was therefore obtained from the animals by ultrasound-guided cystocentesis at the 0, 3, 6, 12, 24 and 48-h study points. The CFU/ml number of the sample was determined and samples were considered positive for bacteriuria if there was growth (> 10^5^ cfu/ml) (Table 1). All study animals received antibiotic prophylactic treatment with enrofloxacin on the previous day and 2 days after every study phase.

**Table 1:** Early bacteriuria

Group/% animals with bacteriuria	0 h	1 h	3 h	6 h	12 h	24 h	48 h
Group-I	0	50	58.3	100*	100*	100	100
Group-II	0	41.6	50	50*	50*	66.6	100

*p < 0.05, Group-I versus Group-II. 6 h and 12 h

### Follow-ups

Follow-ups were performed at 1 (Phase-II), 3 (Phase-III) and 6 weeks (Phase-IV), which correspond to ureteral stent removal in Group-I, and at 8 (Phase-V) and 12 weeks (Phase-VI), using the same diagnostic procedures described above (Table 2).

**Table 2:** Results summary

	Groups	Baseline	1 weeks	3 weeks	6 weeks	8 weeks	12 weeks	20 weeks	Significant
Ultrasound Mode B (SFU score) (G_0_-G_4_)	G-I	G_0_-12	G_0_-1	G_0_-0	G_0_-1	G_0_-6	G_0_-12	G_0_-11	3 weeks*
			G_1_-4	G_1_-5	G_1_-6	G_1_-5		G_1_-1	
			G_2_-5	G_2_-7	G_2_-2	G_2_-1			
			G_3_-2		G_3_-3				
	G-II	G_0_-12	G_0_-2	G_0_-3	G_0_-3	G_0_-12	G_0_-10	G_0_-12	
			G_1_-5	G_1_-7	G_1_-7		G_1_-2		
			G_2_-4	G_2_-2	G_2_-2				
			G_3_-1						
Proximal ureter (diameter. mm)	G-I	6.7 ± 0.5	24.6 ± 3.0	20.8 ± 1.4	16.3 ± 1.2	13.1 ± 1.8	10.9 ± 0.7	8.8 ± 1.2	
	G-II	5.9 ± 0.6	13.7 ± 3.0	16.1 ± 1.3	17.6 ± 1.3	14.7 ± 1.8	12.5 ± 0.7	10.1 ± 1.2	
Bacteriuria (Animals)	G-I	0	2	2	6	N.A	N.A	N.A	
	G-II	0	3	5	8	N.A	N.A	N.A	
Migration	G-I	N.A	2	2	0	N.A	N.A	N.A	
	G-II	N.A	2	3	0	N.A	N.A	N.A	
VUR Score	G-I	G_0_-12	G_0_-10	G_0_-7	G_0_-4	G_0_-10	G_0_-10	G_0_-11	3–6 weeks*
			G_1_-2	G_1_-5	G_1_-6	G_1_-2	G_1_-2	G_1_-1	
					G_2_-2				
	G-II	G_0_-12	G_0_-12	G_0_-12	G_0_-12	G_0_-12	G_0_-12	G_0_-12	
Ureteral orifice Score (UOScore)	G-I	UO_0_-12	UO_0_-1	UO_0_-0	UO_0_-0	UO_0_-3	UO_0_-8	UO_0_-10	1–3–6–8 weeks*
			UO_1_-4	UO_1_-2	UO_1_-4	UO_1_-5	UO_1_-2	UO_1_-2	
			UO_2_-5	UO_2_-8	UO_2_-5	UO_2_-3	UO_2_-2	UO_2_-0	
			UO_3_-2	UO_3_-2	UO_3_-3	UO_3_-1	UO_3_-0	UO_3_-0	
	G-II	UO_0_-12	UO_0_-11	UO_0_-11	UO_0_-12	UO_0_-12	UO_0_-12	UO_0_-12	
			UO_1_-1	UO_1_-1					
Ureteral peristalsis (% animals)	G-I	100	0	0	0	100	100	100	1–3–6 weeks*
	G-II	100	66.6	58.3	75	100	100	100	

*p < 0.005

### Phase VII: Final follow-up: imaging and pathological assessment

The final follow-up was performed at 20 weeks and included ultrasonography, cystoscopy, ureteroscopy and contrast fluoroscopy assessment. The animal study was completed by removing the urinary tract *en bloc* for blind pathological analysis. Histological slices were obtained from proximal ureters, the ureteral segment where the distal end of BraidStent®-H was placed, and ureteral orifices/bladder trigone. A validated healing score was used, and each parameter was graded according to the following scores: 0: no histopathological changes; 1: mild; 2: moderate; 3: severe [3, 6, 12] (Table 3).

**Table 3:** Histological score

Group/ureteral segment	Urothelial resurfacing	Mural inflammation	Lamina propia fibrosis	Integrity of muscular layer	Serosal alterations	0–3
Group-I-Upper ureter	0.85	0.77	0.31	1.31	0.15	0.67 ± 0.41
Group-II-Upper ureter	0.85	0.54	0.08	1.08	0	0.51 ± 0.42
Group-I-UVJ	2.00	1.38*	0.85	0.69	0	0.98 ± 0.67
Group-II-UVJ	1.01	0.62*	0.77	0.46	0	0.57 ± 0.33

*p < 0.05, Group-I-UVJ versus Group-II-UVJ. Mural inflammation

### Statistical analysis

SPSS 22.0 program was used for statistical analysis. To determine the sample size, a hypothesis contrast was performed (the means were compared); a total of 12 animals per group were obtained (0.05 level of significance and 90% statistical power). Normal distribution of variables was confirmed using the Kolmogorov–Smirnov test. Parametric variables were assessed using repeated measures analysis of variance. Categorical variables are expressed as percentages. Comparison between groups of categorical variables were analysed using Fisher’s exact test, and evolution over the course of the study was analysed with McNemar’s test.

## Results

### Phase I: Baseline studies and ureteral stenting

Bacteriuria determinations during the first 48 h shown statistical significance between groups was found at 6 and 12 h, which shows a bacteriuria delaying effect in Group-II caused by the heparin coating. Positive bacteriuria did not reach 100% of animals in Group-II until 48 h, compared with Group-I, in which it appeared at 6 h (Table 1).

No animals showed alterations in the urinary tract or bacteriuria in basal studies. No complications arose during stent placement in any of the groups (Table 2).

### Phase II

A ureteral non-obstructive polypoid growth at the distal end of BraidStent®-H was found in 100% of Group-II animals. Ureteral peristalsis was inhibited in Group-I, and 66.6% of animals in Group-II maintained peristalsis at the distal ureter with statistical significance. Significance was also observed in the evolution of tissue damage at the bladder trigone between groups assessed by UOScore. The most severe damage was found in Group-I (Table 2).

### Phase III

Mild urothelial polypoid growth remained in 100% of animals in Group-II at the distal end of the stent. Overall, 27% of BraidStent®-H showed macroscopic partial degradation, especially of the PGA polymer. Small fragments in the urinary bladder and decolouration of BraidStent®-H components were observed. A higher degree of hydroureteronephrosis in Group-I was noted in this follow-up. No statistical significance regarding stent migration between groups was found; however, this was observed regarding UOScore assessment, and greater damage was found in Group-I.

### Phase IV

**Fig. 2:**
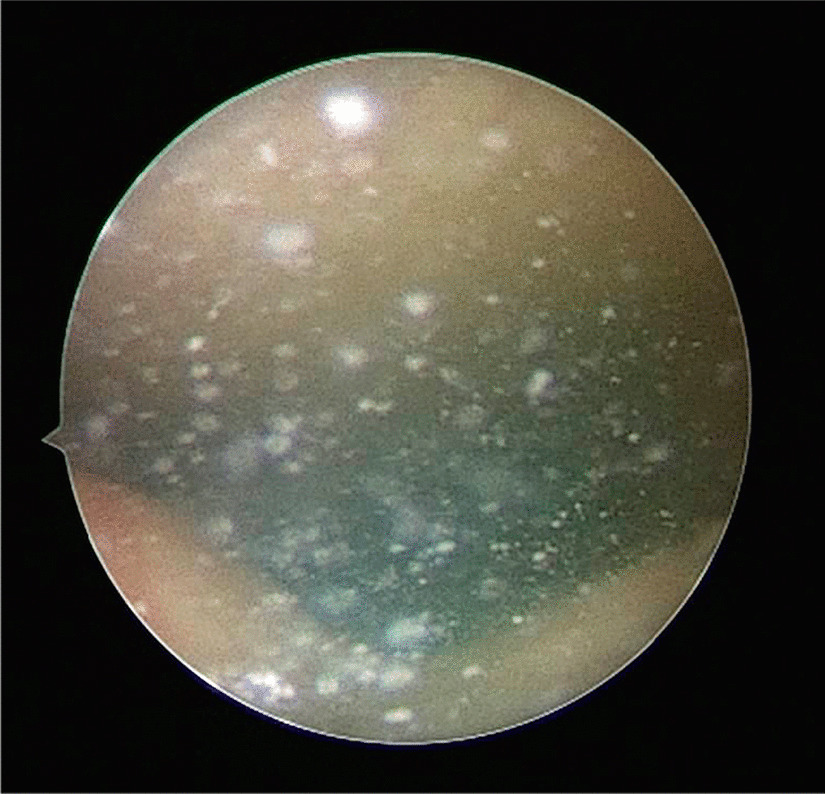
Cystoscopic view of the floating tiny BraidStent®-H fragments at 6 weeks

Overall, 91.7% of BraidStent®-H were completely degraded (Fig. 2). Non-obstructive polypoid growth associated with the BraidStent®-H distal end disappeared. The only exception was the stent, which had not yet fully degraded, 8.3%.

### Phase V

The only partially degraded BraidStent®-H completed degradation and its fragments were eliminated in urine. All study animals recovered ureteral peristalsis. No ureteroscopic evidence of the polypoid growth, found during the early phases in Group-II, was observed. Overall, 100% of animals in Group-II and 50% in Group-I did not show any dilatation of the upper urinary tract after nephrosonographic assessment. Although 2 weeks passed after double pigtail stent removal, statistical significance between groups was observed; with Group-I showed greater macroscopic vesical damage.

### Phase VI

Ureteral internal diameters, hydronephrosis degree and ureteral peristalsis did not show any statistical significance after 12 weeks in both groups versus baselines values. Nevertheless, statistical significance regarding the assessment of urothelial damage at vesical level between groups was still observed.

### End-of-study evaluation

The final follow-up showed the recovery of basal values in all parameters assessed, except for one animal in Group-I, which showed low degree VUR. Statistical significance was only found in lamina propria inflammation of UVJ between groups, after pathological assessment of the ureteral wall and UVJ; Group-I showed greater histological damage (Table 3). The evaluation of the location of the distal end of the BraidStent®-H showed no changes in the ureteral wall.

## Discussion

In the search for an ideal ureteral stent, its biodegradation ability to avoid a new procedure for removal is one of the requirements which have not yet been achieved. BUS not only avoid removal, but also have a series of important advantages, such as the reduction of patient anxiety, since they know they will not need further outpatient cystoscopy stent removal; being cost-saving for the health system; avoiding anaesthetic procedures in paediatric patients; and, last but not least, avoiding the syndrome of forgotten stent [3, 13, 14].

Therefore, several research groups have assessed different BUS prototypes in animal model in the last decade [4, 5, 15, 16]. This research obtained encouraging, but inconclusive results, mainly due to the limitations of experimental studies and the scarce number of animals involved in the studies. A research group with a higher number of BUS-assessed animal models, 36 stents, and with a longer follow-up period (5 months) evaluated the BraidStent® [3, 6, 12]. This biodegradable intraureteral stent shows a limitation due to its high asymptomatic bacteriuria rate, which is up to 41.6%. However, it has shown a predictable degradation rate and non-obstructive stent fragmentation.

The use of heparin for stent coating and to prevent bacterial adhesion, avoiding biofilm and incrustation formation, was previously assessed in depth [7, 8, 17]. Heparin is useful because it has a high negative charge which provides it an important bacterial anti-adhesive effect. However, in an in vitro study, Lange et al. [9] found that the heparin coating did not decrease bacterial adhesion, although they initially obtained encouraging results.

Comparative results of the current experimental study confirm that the heparin coating shows statistical significance regarding bacteriuria between groups, from the sixth hour post-stenting. Asymptomatic bacteriuria did not affect 100% of animals in the BraidStent®-H group until 48 h compared with standard ureteral stents, which showed positive bacteriuria at 6 h. Therefore, heparin coating shows differences compared to a standard stent, although it does not have a significant antibacterial effect, and it also fulfils the purpose for which it was used in this study: to avoid bacterial colonisation at stent placement. BraidStent®-H, unlike other heparin-coated stents, is designed so that the coating disappears within the first 72 h and does not affect the stent degradation rate [7, 8]. In previous in vitro and in in vivo studies by other researchers’ evaluated stents incorporate heparin inside of the polyurethane matrix rather than a heparin coating [7, 8, 17]. Consequently, heparin release is scarce and may hamper the prevention of bacterial adhesion [17]. This is an important difference to our coating technique, as our heparin coating is designed to be released in the early days of stenting. Research into a coating that prevents bacterial adhesion is extremely complicated, since bacteria utilise a multitude of mechanisms for surfaces attaching, which are specific to bacterial species [9].

The intermediate follow-ups in Group-II at 1, 3 and 6 weeks were disappointed (25% in the first week, 41.6% in the third week and 66.6% in the sixth week, with a 44.4% study average), despite the initial bacteriuria rate decreasing. This means a setback of the heparin coating in this formulation to reduce bacteriuria in the long-term. Different factors need to be considered in order to discuss these results. Firstly, it is necessary to compare them with other results described in clinical trials evaluating 30–60 days of standard ureteral stenting: 21.9% [18], 24.3% [10], 23.6% [19], 28% [20], 45.8% [21]. In this regard, our positive asymptomatic bacteriuria rate was within an elevated range: BraidStent®-H values were 1.5–2 times higher than the bacteriuria rate, except for in the study by Shabeena (45.8%) [21]. Nevertheless, a second, very important factor must be considered in order to critically assess these unsatisfactory results. When assessing a BUS, stent fragments carry the biofilm which covers all ureteral stents and causes the bacteria embedded in the biofilm to become free or results in planktonic bacteria in the urine by breaking the integrity of the biofilm. Consequently, if our results are compared with scientific literature on the bacterial colonisation of ureteral stents with a similar stenting time (29.4% [22], 42% [20], 46.2% [21], 58.6% [23], 82.9% [10], 98.5% [20], 100% [24]), experimental values are not as high as they initially seemed to be, since they take into account the continuous fragmentation of the biofilm layer. One final factor could explain these disappointing, asymptomatic bacteriuria results: all study animals were females. Several authors have reported that women can double the likelihood of ureteral stent colonisation, due to the length of their urethra [25]. Gender is not considered in any of the percentages described in the above-mentioned clinical trials.

However, is obviously necessary to study the control of asymptomatic bacteriuria in BUS in greater depth, in order to avoid possible urinary tract infections (UTI) with these new ureteral stent designs. In previous experimental studies assessing other BUS designs, high variability and methodologies were found. Thus, there are BUS in which bacteriuria is not evaluated [4], or others in which the placement of the stent is by cystotomy, which produces a great bias to evaluate the bacterial colonization of the BUS [5, 15, 26]. Others assess urinary infection only by determining WBCs in urine, but this assesment is not comparable to the appropriate protocol for assessing colonisation, such as measurement of bacteriuria, urine culture or stent culture [5, 27, 28]. In two studies of the same research group, researchers found that after evaluating their BUS, Uriprene®, in a pig model, different results were found with higher positive urine cultures in biostable stents in one study and in the other slightly higher positive cultures in BUS [16, 29]. Therefore, at present, due to high variability in the studies and designs evaluated, it is not possible to conclude whether BUSs favour urinary bacterial contamination.

With regard to the other properties evaluated in the comparative study, BraidStent®-H shows a similar passive dilatation at the proximal ureter, although there is a less hydronephrotic effect, with significance noted after 3 weeks. We have shown that BraidStent®-H will predictably decrease patient discomfort due to its design, as it keeps ureteral peristalsis below the stent, with significance in the first, third and sixth weeks. Other factors that predict less discomfort in patients are the absence of VUR and less macroscopic damage at the bladder trigone (UOScore), as shown by the statistical significance of the study [2]. The preservation of peristalsis, absence of VUR and absence of ureteral stent in the distal ureter should avoid the onset of ureteral spasms in patients at the ureteral segment with a higher density of nerve fibers [30]. On the other hand, as shown by the results of UOScore and pathological UVJ assessment between groups, and as also demonstrated by Yoshida et al. [31] in a study of patients, an intra-ureteral stent was associated with less bladder discomfort than a standard ureteral stent.

Degradation rate control is one of the major challenges to the clinical use of BUS. Our study shows the BraidStent®-H unlike other evaluated BUS showed a predictable degradation rate despite its heparin coating [4, 5, 27, 29]. Furthermore, degradation fragments were easily eliminated, finding that in others BUS experimental studies triggered problems, with small fragments in the upper tract and embedded in the ureteral wall [5, 26]. The positive results of BraidStent®-H in this regard are due to its braided design, which produced very small fragments, as well as the choice of polymers with different degradation rates, which allowed the progressive stent hydrolysis.

The current weaknesses of the BraidStent®-H are linked to asymptomatic bacteriuria rates and migration rates. We only found a slight dislodgement of the proximal end. However, this provided information on a small structural weakness in the BraidStent®-H proximal tip. Since BraidStent®-H is an intraureteral stent, dislodgment in placement or early migration implies a technical challenge for urologists.

Study limitations are intrinsic to the animal model, as it is obviously not possible to develop any kind of USSQ questionnaire in order to assess degree of discomfort in study subjects. On the other hand, SVCUG was used for VUR assessment instead of voiding cystourethrography. Finally, it is difficult to evaluate whether daily changes of pH in humans can modify the BraidStent-H degradation rate, as animals follow a controlled diet. Despite the encouraging results, further studies will be required to determine whether the use and safety of BUS with changing urinary pH conditions in patients allows adequate control in the rate of BUS degradation.

## Conclusions

The heparin coating of BraidStent® allows an early decrease in urinary bacterial colonization, but its effectiveness is low at the long term. The heparin coating did not affect the scheduled degradation rate or the size of the stent fragments. The BraidStent®-H avoids the side effects associated with current ureteral stents, and should therefore cause less discomfort to patients. However, further studies will be needed to improve the anchoring system and reduce the risk of bacterial colonisation.

## Data Availability

All data generated or analyzed during this study are included in this published article.

## Abbreviations

VUR: Vesico-ureteral reflux
BUS: Biodegradable ureteral stent
UOScore: Ureteral orifice score
SVCUG: Simulated voiding cystourethrography
EU: Excretory urography
UPJ: Ureteropelvic junction
UVJ: Ureterovesical junction
PGA: Poliglicolic acid
CFU: Colony forming unit
UTI: Urinary tract infection
WBCs: White blood cells
